# Health outcomes and healthcare resource utilization among Veterans with stage IV non-small cell lung cancer treated with second-line chemotherapy versus immunotherapy

**DOI:** 10.1371/journal.pone.0282020

**Published:** 2023-02-21

**Authors:** Christina D. Williams, Mina A. Allo, Lin Gu, Vishal Vashistha, Ashlyn Press, Michael Kelley

**Affiliations:** 1 Cooperative Studies Program Epidemiology Center-Durham, Durham Veterans Affairs Health Care System, Durham, North Carolina, United States of America; 2 Medical Oncology, Department of Medicine, Duke University, Durham, North Carolina, United States of America; 3 Bristol-Myers Squibb Company, US Health Economics and Outcomes Research, Princeton, New Jersey, United States of America; 4 Duke Cancer Institute, Biostatistics Shared Resource, Duke University, Durham, North Carolina, United States of America; 5 Hematologic Malignancies and Cellular Therapy, Department of Medicine, Duke University, Durham, North Carolina, United States of America; 6 Division of Hematology-Oncology, Medical Service, Durham Veterans Affairs Health Care System, Durham, North Carolina, United States of America; Northwestern University Feinberg School of Medicine, UNITED STATES

## Abstract

**Background:**

Until recently, multi-agent chemotherapy (CT) was the standard of care for patients with advanced non-small cell lung cancer (NSCLC). Clinical trials have confirmed benefits in overall survival (OS) and progression-free survival with immunotherapy (IO) compared to CT. This study compares real-world treatment patterns and outcomes between CT and IO administrations in second-line (2L) settings for patients with stage IV NSCLC.

**Materials and methods:**

This retrospective study included patients in the United States Department of Veterans Affairs healthcare system diagnosed with stage IV NSCLC during 2012–2017 and receiving IO or CT in the 2L. Patient demographics and clinical characteristics, healthcare resource utilization (HCRU), and adverse events (AEs) were compared between treatment groups. Logistic regression was used to examine differences in baseline characteristics between groups, and inverse probability weighting multivariable Cox proportional hazard regression was used to analyze OS.

**Results:**

Among 4,609 Veterans who received first-line (1L) therapy for stage IV NSCLC, 96% received 1L CT alone. A total of 1,630 (35%) were administered 2L systemic therapy, with 695 (43%) receiving IO and 935 (57%) receiving CT. Median age was 67 years (IO group) and 65 years (CT group); most patients were male (97%) and white (76–77%). Patients administered 2L IO had a higher Charlson Comorbidity Index than those administered CT (*p* = 0.0002). 2L IO was associated with significantly longer OS compared with CT (hazard ratio 0.84, 95% CI 0.75–0.94). IO was more frequently prescribed during the study period (p < 0.0001). No difference in rate of hospitalizations was observed between the two groups.

**Conclusions:**

Overall, the proportion of advanced NSCLC patients receiving 2L systemic therapy is low. Among patients treated with 1L CT and without IO contraindications, 2L IO should be considered, as this supports potential benefit of IO for advanced NSCLC. The increasing availability and indications for IO will likely increase the administration of 2L therapy to NSCLC patients.

## Introduction

Lung cancer is the second most common cancer and the leading cause of cancer-related death among men and women in the United States (US). Non-small cell lung cancer (NSCLC) is the predominant histologic subtype, accounting for approximately 87% of lung cancer cases and typically diagnosed at an advanced stage. Before the era of immunotherapy (IO), the 5-year survival rate for patients with advanced NSCLC was only 5%, and even with modest improvements remain less than 10% [1, 16].

Before 2015, the standard of care for metastatic NSCLC was platinum-based chemotherapy (CT). However, in recent years, the therapeutic landscape has changed rapidly with the development of a range of new treatments. In addition to targeted therapies for patients with actionable gene mutations, a number of immune checkpoint inhibitors have now been approved for use in both first-line (1L) and second-line (2L) treatment settings for patients with metastatic NSCLC regardless of the results of biomarker testing [2]. IO therapies have been demonstrated to significantly improve progression-free survival (PFS) and overall survival (OS) in comparison to CT [3–7]. Studies have also shown that IO has an acceptable toxicity profile in comparison to other therapies [8, 9].

Although the clinical trial results for IO therapies are compelling, real-world studies of treatment effectiveness, safety, healthcare resource utilization (HCRU), and patterns of use among patients with metastatic NSCLC in clinical practice are crucial to understand the medical and economic impacts of IO therapies and to identify unmet needs. Clinical trials are highly selective, and few adult cancer patients are eligible for clinical trials. For those who are candidates, they tend to be relatively young and healthy, in contrast to the mostly elderly general population of patients with metastatic NSCLC seen in general oncology practice [8]. For example, approximately 70% of patients with NSCLC would fail to meet the inclusion criteria for the clinical trials used to approve these treatments [10, 11]. Moreover, in comparison to patients in clinical trials, patients with NSCLC treated with programmed cell death protein 1 inhibitors within community cancer care clinics were older when treatment was initiated and had reduced OS [12, 13]. Such findings suggest that the benefits of IO may not be as pronounced in comparison to CT in a real-world setting.

Given that IO has been approved for patients in the 2L setting for years regardless of the results of precision testing, a comprehensive retrospective comparison between those treated with IO and CT can now be completed. Consequently, this study evaluated the treatment patterns, survival outcomes, HCRU, and adverse events (AEs) among Veterans with metastatic NSCLC treated within cancer practices of the U.S. Department of Veterans Affairs (VA) Healthcare System.

## Materials and methods

### Patient selection

**Fig 1:**
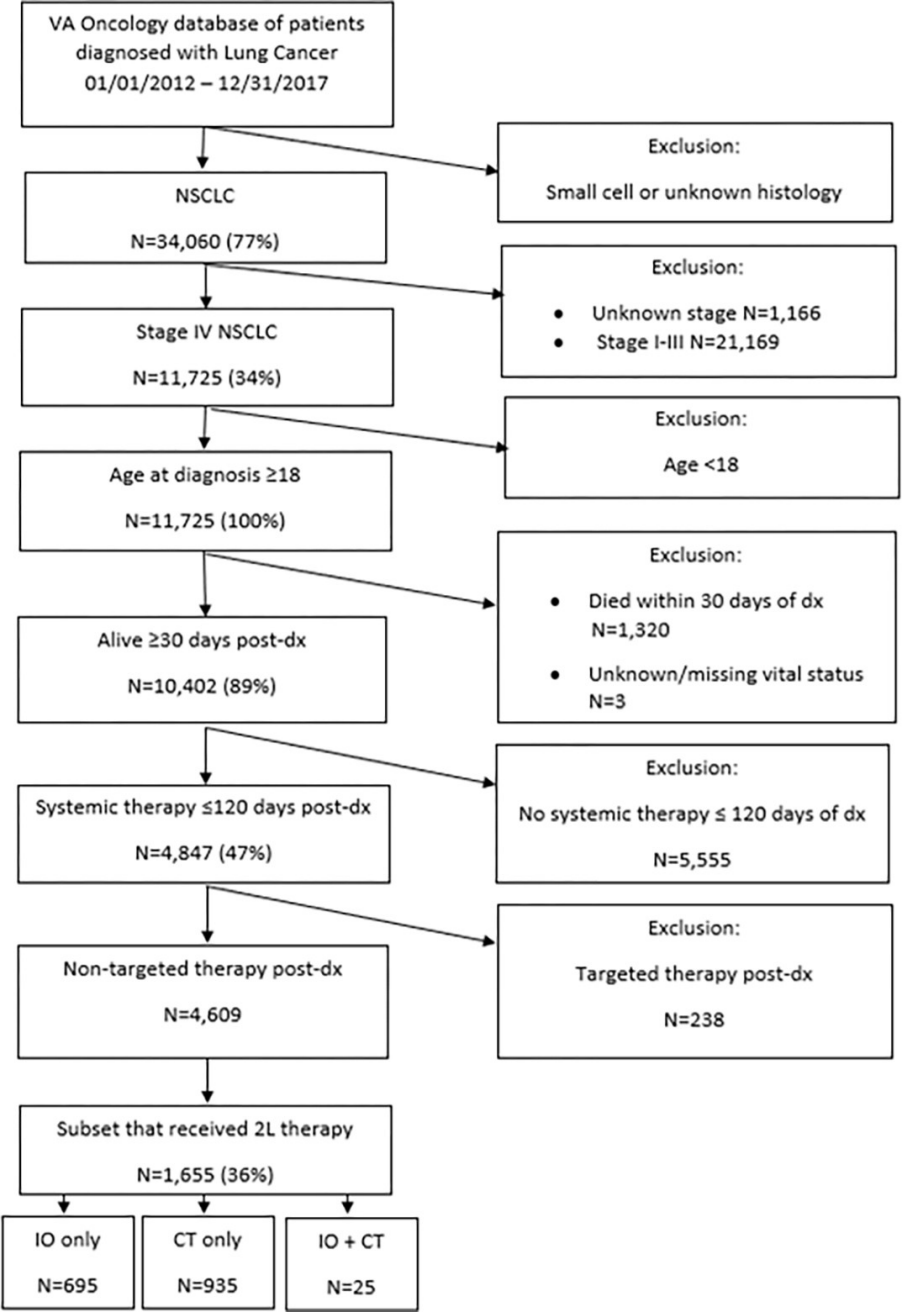
Patient selection flowchart. 2L: second-line; CT: chemotherapy; dx: diagnosis; IO: immunotherapy; NSCLC: non-small cell lung cancer; VA: Veterans Affairs.

This retrospective study included patients diagnosed with Stage IV NSCLC between January 1, 2012 and December 31, 2017, as identified in the VA’s Corporate Data Warehouse (CDW) Oncology database (Fig 1). Patients were at least 18 years old, received systemic therapy within 120 days of the diagnosis date, and subsequent (2L) therapy. Patients were excluded if they had received ALK or epidermal growth factor receptor (EGFR) targeted therapy or died within 30 days of diagnosis. Patients were followed from diagnosis until death from any cause or at least one year from diagnosis to the last date of healthcare utilization before the end of the study period, which was April 18, 2019. This is a retrospective study using VA’s administrative and EHR data within the VA Corporate Data Warehouse (CDW). Data were not anonymized because patient identifiers were necessary to link data from various sources within the VA CDW. A waiver of informed consent and HIPAA authorization was requested and approved through the Durham VA Health Care System’s Institutional Review Board.

### Data sources and measures

The VA CDW aggregates data from the universal electronic medical record and includes discrete data on patient demographics, procedures, diagnoses, clinical encounters, medications, vital signs and vital status. The oncology database within CDW is derived from the VA Cancer Registry System and includes data for patients diagnosed with and/or treated for cancer in the VA healthcare system along with information on their tumor and other characteristics. Therefore, we used this specific database to obtain tumor stage, histology, diagnosis date, smoking history, and demographic data (age, race, marital status, geographic region) at the time of diagnosis. The CDW pharmacy data were accessed to capture utilization of CT and IO agents in the 2L setting. The start of 2L therapy was defined as administration of a new drug on or after 21 days of initial therapy or after 21 days of no treatment. HCRU was defined as the number of outpatient visits and hospitalizations, and length of stay among those hospitalized. HCRU was evaluated from the start of 2L therapy to the start of subsequent line of therapy or 90 days after the end date of 2L therapy. The CDW vital status data allowed us to characterize OS as the time from the start of 2L therapy to death from any cause. Patients surviving until the end of the study period were censored and their follow-up time evaluated from the date of diagnosis until last HCRU. AEs were defined on the basis of the presence of International Classification of Diseases-9/10 (ICD-9/10) codes for common IO and CT-related AEs documented as primary or secondary diagnoses on inpatient and outpatient encounters between the initiation of 2L therapy and start of subsequent line of therapy, if received, or 90 days after last treatment ended. Common AEs of interest were rash/inflammatory dermatitis, colitis/enterocolitis, hepatitis, hypophysitis/hypopituitarism, hypothyroidism, pneumonitis, dyspnea, anemia, atypical pneumonia, fatigue, nausea/vomiting, neuropathy, and fever with neutropenia. Inpatient and outpatient encounters were also used to compute each patient’s Charlson Comorbidity Index (CCI), which captured diagnoses within 1 year of the lung cancer diagnosis.

### Statistical analyses

Patients were separated into two treatment groups for comparison: treatment with CT only versus IO only in the 2L. The proportion of patients receiving each therapy during the study period and the distribution of treatment groups over the 6-year study period were evaluated. The following descriptive statistics were used to summarize patient characteristics: counts and percentages for categorical variables, and mean (standard deviation [SD]) or median (range) for continuous variables. All demographic and clinical characteristics were stratified by treatment group and compared using chi-square or Fisher’s exact test for categorical variables and 2-sided t test or Wilcoxon rank sum test for continuous variables, as appropriate. Logistic regression analyses were conducted to evaluate the association between patient characteristics and type of treatment. OS was analyzed using the Kaplan–Meier method to estimate median, 6-month, and 12-month survival and associated 95% confidence Intervals (CI), and survival curves were stratified by treatment group (CT only, IO only) and compared using the log-rank test. Stabilized inverse probability weighting (IPW) multivariable Cox proportional hazards model was used to evaluate the impact of treatment (reference: CT only) and other demographic and clinical characteristics on OS. Logistic and mixed linear regression models were used to evaluate the association between treatment (reference: CT only) and HCRU and AE rates (i.e. a binary variable for each AE and one global measure for any AE). Multivariable logistic and Cox regression model effects were estimated using odds ratios (OR) and hazard ratios (HR), respectively, and corresponding 95% CIs. All models were adjusted for age, race, region, histology, smoking and marital status at diagnosis, prior radiation or surgical treatment, and comorbidities. A P value <0.05 was considered statistically significant, two-sided P values were reported, and all analyses were performed using Statistical Analysis Software® (SAS) version 9.4 (SAS Institute Inc., Cary, NC, USA).

This study was approved by the Durham VA Health Care System’s Institutional Review Board.

## Results

### Patient characteristics

A total of 4,609 patients were administered 1L therapy, with 4,426 (96%) receiving CT only, 156 (3%) receiving IO only, and 27 (1%) receiving IO + CT. Of these, 1,630 (35%) patients were selected for inclusion of 2L analysis; 695 (43%) were treated with IO and 935 (57%) were treated with CT (Fig 1). The CT group included patients receiving single-agent or multi-agent regimens, whereas the IO group solely included patients receiving single-agent immune checkpoint inhibitors; no patients had received dual IO regimens during the study period. The median age was 67 years in the IO group and 65 years in the CT group (Table 1). Most patients were male (96.7% in the IO group and 96.9% in the CT group), as expected, and white (76.3% in the IO group and 77.4% in the CT group). Most patients were resident in the South or Midwest of the US (IO group: 34.5% and 32.2%, respectively; CT group: 37.9% and 25.7%). Over 95% of patients in both treatment groups were current or former smokers, and most presented with adenocarcinoma histology (IO group: 58.6%; CT group: 57.4%). More patients in the IO group than in the CT group had a CCI ≥3 (36.8% and 27.2%, respectively). IO patients had a median drug exposure of 85 days and mean of 186 days, while CT patients had median drug exposure of 53 days and mean of 99 days.

**Table 1:** Patient demographic and clinical characteristics by 2L treatment.

	IO only (N = 695)	CT only (N = 935)	P-value
**Year of diagnosis**			
2012	3 (0.4%)	270 (28.9%)	< .0001^1^
2013	22 (3.2%)	268 (28.7%)	
2014	66 (9.5%)	211 (22.6%)	
2015	218 (31.4%)	80 (8.6%)	
2016	247 (35.5%)	50 (5.3%)	
2017	139 (20.0%)	56 (6.0%)	
**Year of initial 2L administration**			
2012	2 (0.3%)	232 (24.8%)	< .0001^1^
2013	19 (2.7%)	278 (29.7%)	
2014	51 (7.3%)	217 (23.2%)	
2015	203 (29.2%)	90 (9.6%)	
2016	246 (35.4%)	55 (5.9%)	
2017	158 (22.7%)	59 (6.3%)	
2018	16 (2.3%)	4 (0.4%)	
**Age**			
Median (range)	67 (35, 89)	65 (29, 92)	0.0005^2^
< 50	9 (1.3%)	14 (1.5%)	0.0007^1^
50–64	227 (32.7%)	396 (42.4%)	
65–74	369 (53.1%)	411 (44.0%)	
> 74	90 (12.9%)	114 (12.2%)	
**Sex**			
Female	23 (3.3%)	29 (3.1%)	0.8134^1^
Male	672 (96.7%)	906 (96.9%)	
**Race**			
White	530 (76.3%)	724 (77.4%)	0.7636^1^
Black	146 (21.0%)	186 (19.9%)	
Other	11 (1.6%)	11 (1.2%)	
Unknown	8 (1.2%)	14 (1.5%)	
**Ethnicity**			
Hispanic	10 (1.4%)	10 (1.1%)	0.7635^1^
Non-Hispanic	683 (98.3%)	923 (98.7%)	
Unknown	2 (0.3%)	2 (0.2%)	
**Marital status at diagnosis**			
Married	312 (44.9%)	411 (44.0%)	0.4730^1^
Unmarried	378 (54.4%)	521 (55.7%)	
Unknown	5 (0.7%)	3 (0.3%)	
**Region**			
Northeast	91 (13.1%)	158 (16.9%)	0.0113^1^
South	240 (34.5%)	354 (37.9%)	
Midwest	224 (32.2%)	240 (25.7%)	
West	140 (20.1%)	183 (19.6%)	
**Smoking history**			
Current/former smoker	665 (95.7%)	891 (95.3%)	0.1457^1^
Non-smoker	23 (3.3%)	24 (2.6%)	
Unknown/missing	7 (1.0%)	20 (2.1%)	
**Histology**			
Squamous	227 (32.7%)	289 (30.9%)	0.1615^1^
Adenocarcinoma	407 (58.6%)	537 (57.4%)	
Other	61 (8.8%)	109 (11.7%)	
**Radiation**			
No	431 (62.0%)	599 (64.1%)	0.3163^1^
Yes	261 (37.6%)	335 (35.8%)	
Unknown	3 (0.4%)	1 (0.1%)	
**Surgery**			
No	682 (98.1%)	922 (98.6%)	0.6155^1^
Yes	11 (1.6%)	12 (1.3%)	
Unknown	2 (0.3%)	1 (0.1%)	
**Charlson Comorbidity Index**			
0	201 (28.9%)	311 (33.3%)	0.0002^1^
1–2	238 (34.2%)	370 (39.6%)	
≥3	256 (36.8%)	254 (27.2%)	
**Drug Exposure**			
** Median (range)**	85 (233)	53 (153)	
** Mean (SD)**	186 (1–1211)	99 (1–1767)	

^1^Chi-square p-value

^2^Wilcoxon rank sum test

2L: second-line; CT: chemotherapy (monotherapy or combination therapy); IO: immunotherapy monotherapy

### Predictors of receipt of IO

The proportion of patients receiving 2L IO increased over time, with some decrease in 2017, while the proportion of patients receiving 2L CT decreased significantly over time (Table 1). Patients treated with 1L IO were less likely to receive IO as 2L therapy, in comparison to patients being treated with 1L CT (OR 0.04, 95% CI 0.01–0.28). The odds of older patients receiving 2L IO were higher than 2L CT (OR 1.20 per 10 years, 95% CI 1.04–1.38). Patients living in the Midwest region were observed to have higher odds of receiving 2L IO than 2L CT than those in the Northeast region (OR 1.69, 95% CI 1.22–2.33). Patients with higher CCI were more likely to receive 2L IO (OR 1.49, 95% CI 1.15–1.93) (Table 2).

**Table 2:** Multivariable logistic regression to assess association between patient characteristics and receipt of 2L IO vs CT.

	OR (95% CI) (IO vs CT)	P-value
**First-line therapy (IO vs CT)**		0.0011
** IO**	0.04 (0.01–0.28)	
**Age (unit = 10 years)**	1.20 (1.04–1.38)	0.0116
**Race (ref = White)**		0.5943
** Black**	1.14 (0.88–1.48)	
** Other**	0.99 (0.53–1.87)	
**Marital status at diagnosis (ref = Unmarried)**		0.9196
** Married**	0.99 (0.80–1.22)	
** Unknown**	1.37 (0.28–6.73)	
**Region (ref = Northeast)**		0.0052
** South**	1.17 (0.86–1.60)	
** Midwest**	1.69 (1.22–2.33)	
** West**	1.33 (0.94–1.88)	
**Smoking history (ref = Current/former)**		0.9118
** Non-smoker/unknown**	0.97 (0.59–1.59)	
**Histology (ref = Adenocarcinoma)**		0.1369
** Squamous**	0.99 (0.79–1.24)	
** Other**	0.71 (0.50–1.00)	
**Prior radiation (ref = No)**		0.2811
** Yes**	1.15 (0.93–1.41)	
** Unknown**	4.33 (0.26–72.16)	
**Prior surgery (ref = No)**		0.6621
** Yes**	1.51 (0.61–3.70)	
** Unknown**	0.77 (0.04–16.54)	
**Charlson Comorbidity Index (ref = 0)**		0.0010
** 1–2**	0.98 (0.76–1.25)	
** ≥3**	1.49 (1.15–1.93)	

2L: second-line; CI: confidence interval; CT: chemotherapy (monotherapy or combination therapy); IO: immunotherapy (monotherapy); OR: odds ratio

### Overall survival

**Fig 2:**
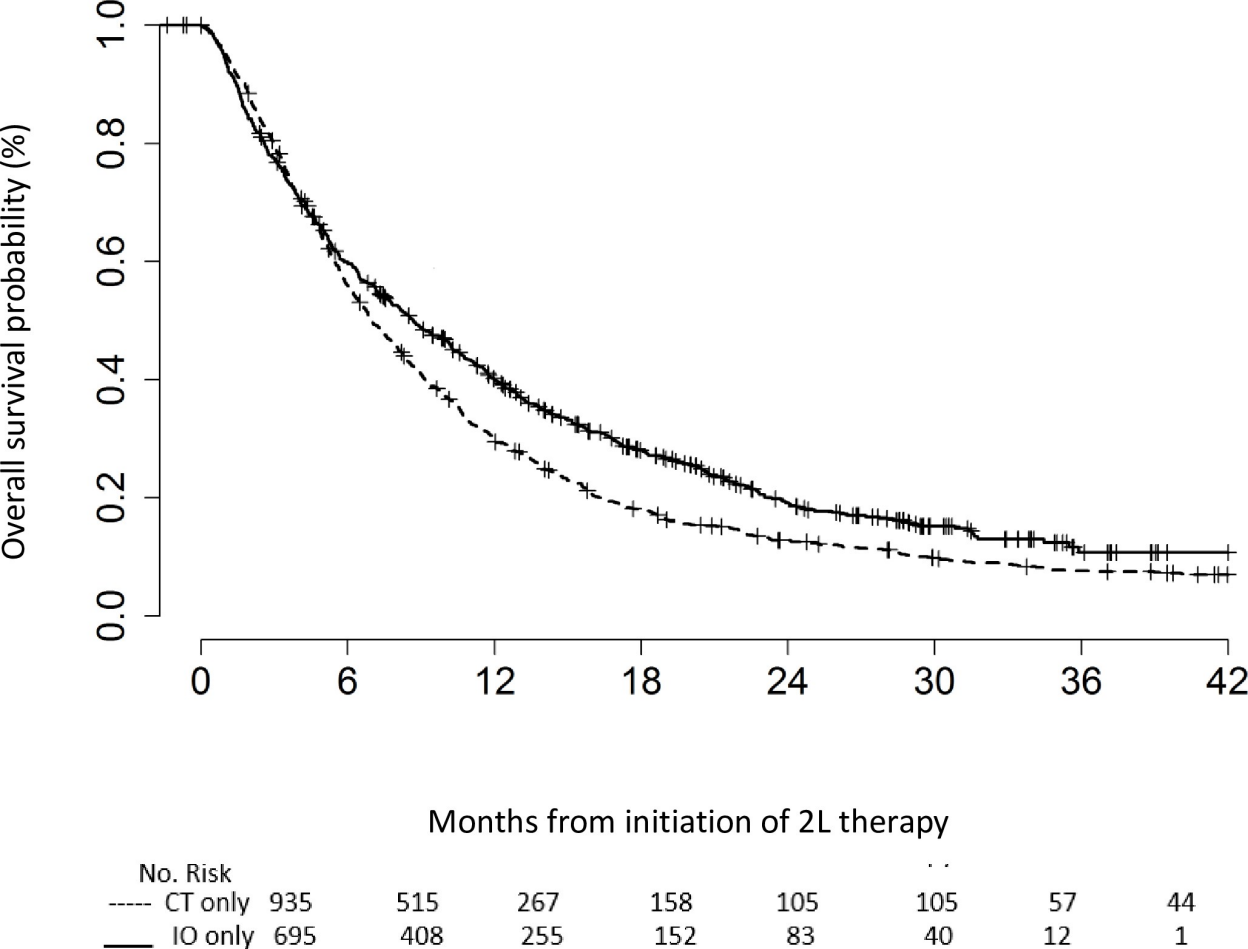
Kaplan–Meier plot of OS for patients who received 2L IO vs CT, from initiation of 2L therapy. 2L: second-line; CI: confidence interval; CT: chemotherapy; Est: estimated; HR: hazard ratio; IO: immunotherapy; OS: overall survival; Tx: treatment.

Median OS from the start of 2L therapy was longer in the IO group than in the CT group (8.7 months vs 7.0 months) (Fig 2). In the multivariable analysis, 2L IO was associated with significantly longer OS than CT (HR 0.84, 95% CI 0.75–0.94, p = 0.0021). Black patients had better survival outcomes than whites (HR 0.84, 95% CI 0.73–0.95). Shorter survival times were observed for patients who received IO vs CT as 1L therapy (HR 1.88, 95% CI 1.07–3.30), underwent prior radiation (OR 1.18, 95% CI 1.06–1.33), and had squamous cell histology (HR 1.14, 95% CI 1.02–1.28) (Table 3).

**Table 3:** Inverse probability weighting Cox proportional hazard regression to evaluate the effect of treatment on OS.

	HR (95% CI) (IO vs CT)	P-value
**First-line therapy (IO vs CT)**	1.88 (1.07–3.30)	0.0286
**Second-line therapy (IO vs CT)**	**0.84 (0.75**–**0.94)**	**0.0021**
**Age (unit = 10 years)**	1.00 (0.99–1.01)	0.6926
**Race (ref = White)**		
** Black**	0.84 (0.73–0.95)	0.0080
** Other**	1.16 (0.81–1.66)	0.4149
**Marriage status (ref = Unmarried)**		
** Married**	1.00 (0.90–1.12)	0.9455
** Unknown**	1.08 (0.45–2.64)	0.8581
**Region (ref = Northeast)**		
** Midwest**	1.02 (0.86–1.21)	0.8423
** South**	0.92 (0.78–1.08)	0.3007
** West**	0.92 (0.77–1.10)	0.3429
**Smoking history (ref = Current/former smoker)**		
** Non-smoker/unknown**	1.00 (0.79–1.28)	0.9848
**Histology (ref = Adenocarcinoma)**		
** Squamous**	1.14 (1.02–1.28)	0.0237
** Other**	0.97 (0.80–1.16)	0.7110
**Prior radiation (ref = No)**		
** Yes**	1.18 (1.06–1.33)	0.0039
** Unknown**	0.73 (0.16–3.31)	0.6882
**Prior surgery (ref = No)**		
** Yes**	0.66 (0.40–1.10)	0.1108
** Unknown**	1.97 (0.67–5.80)	0.2160
**Charlson Comorbidity Index (ref = 0)**		
** 1–2**	0.89 (0.78–1.01)	0.0606
** ≥3**	0.97 (0.85–1.10)	0.6062

CI: confidence interval; CT: chemotherapy; HR: hazard ratio; IO: immunotherapy; OS: overall survival

HR adjusted for all variables in table

### Healthcare resource utilization

HCRU for CT patients and IO patients is shown in Table 4. Patients receiving 2L IO therapy had a mean of 47 overall outpatient visits and those receiving CT has a mean of 31. The mean number of oncology-specific outpatient visits was 14 vs 9 in IO and CT groups, respectively. The proportion of IO patients hospitalized during the study period was 49% compared to 42% among CT patients. Nonetheless, upon multivariable analysis, no significant difference in any HCRU measure was seen between the cohorts.

**Table 4:** Summary of HCRU in 2L patients.

	Descriptive statistics			Odds ratio of IO vs CT	
	IO only (N = 695)	CT only (N = 935)	P-value	Adjusted OR^1^ (95% CI)	P-value
**No. of outpatient visits, n (%)**					
** 0**	7 (1.0)	18 (1.9)	< .0001^2^	0.16 (0.00–5.92) (≥1 vs 0)	0.3193
** 1–20**	199 (28.6)	416 (44.5)			
** 21–60**	307 (44.2)	392 (41.9)			
** >60**	182 (26.2)	109 (11.7)			
**No. of outpatient visits**			< .0001^3^		
** Mean (SD)**	47 (42)	31 (29)			
**No. of oncology outpatient visits, n (%)**					
** 0**	67 (9.6)	106 (11.3)	< .0001^2^	0.91 (0.21–4.00) (≥1 vs 0)	0.8992
** 1–20**	481 (69.2)	755 (80.7)			
** 21–60**	138 (19.9)	72 (7.7)			
** >60**	9 (1.3)	2 (0.2)			
**No. of oncology outpatient visits**			< .0001^3^		
** Mean (SD)**	14 (15)	9 (9)			
**No. of hospitalizations, n (%)**			0.0054^2^		
** 0**	357 (51.4)	545 (58.3)		1.03 (0.46–2.30) (≥1 vs 0)	0.9490
** ≥1**	338 (48.6)	390 (41.7)			
** Range**	1–8	1–7			
** Length of hospital stay (days)**					
** Estimated mean ± SE**	9.1 ± 2.9	9.9 ± 2.9	0.39221^3^		
**30-day admission rate, n (%)**	127 (18.3)	170 (18.2)	0.9622^2^	0.65 (0.20–2.07) (yes vs no)	0.4667
**90-day admission rate, n (%)**	215 (30.9)	285 (30.5)	0.8442^2^	0.68 (0.28–1.62) (yes vs no)	0.3794

2L: second-line; CI: confidence interval; CT: chemotherapy; HCRU: healthcare resource utilization; IO: immunotherapy; OR: odds ratio; SD: standard deviation; SE: standard error

^1^F-test from mixed linear model adjusting for first-line therapy (IO/CT), age, race, region, histology type, smoking status, marital status at diagnosis, prior radiation therapy (yes/no), prior surgical treatment (yes/no), and Charlson Comorbidity Index (0, 1–2, and >2). Variance component was used for covariance structure

^2^Chi-square p-value

^3^t-test

### Adverse events

In the IO group 17.7% of patients had no AEs, compared with 29.6% of CT patients. There was no statistically significant differences in the rates of the following AEs in both treatment groups: rash/inflammatory dermatitis, hepatitis, hypophysitis/hypopituitarism, pneumonitis, anemia, and neuropathy. The rate of common AEs in the IO group were: dyspnea (50%), colitis/enterocolitis (40%), and anemia (28%). Rates of common AEs in the CT group were: anemia (29%), colitis/enterocolitis (26%), and nausea/vomiting (25%) (Table 5).

**Table 5:** AE occurrence by 2L treatment.

	IO only	CT only	Adjusted OR^1^ (95% CI)	P-value
	N (%)	N (%)		
**No. of AEs**				
** None**	123 (17.7)	277 (29.6)	Ref	
** 1–2**	313 (45.0)	366 (39.1)	1.91 (1.46–2.49)	<0.0001
** 3–4**	175 (25.2)	215 (23.0)	1.81 (1.34–2.44)	0.0001
** ≥5**	84 (12.1)	77 (8.2)	2.23 (1.52–3.29)	<0.0001
**Rash/inflammatory dermatitis**	58 (8.3)	63 (6.7)	1.19 (0.81–1.74)	0.3757
**Colitis/enterocolitis**	281 (40.4)	244 (26.1)	1.92 (1.55–2.39)	<0.0001
**Hepatitis**	35 (5.0)	38 (4.1)	1.38 (0.85–2.26)	0.1969
**Hypophysitis/hypopituitarism**	4 (0.6)	1 (0.1)	6.68 (0.71–62.95)	0.0971
**Hypothyroidism**	110 (15.8)	64 (6.8)	2.49 (1.77–3.48)	<0.0001
**Pneumonitis**	25 (3.6)	26 (2.8)	1.31 (0.73–2.33)	0.3658
**Dyspnea**	345 (49.6)	182 (19.5)	4.00 (3.19–5.03)	<0.0001
**Anemia**	193 (27.8)	271 (29.0)	0.90 (0.72–1.13)	0.3871
**Atypical pneumonia**	138 (19.9)	58 (6.2)	3.78 (2.71–5.29)	<0.0001
**Fatigue**	55 (7.9)	160 (17.1)	0.38 (0.27–0.53)	<0.0001
**Nausea/vomiting**	96 (13.8)	236 (25.2)	0.46 (0.35–0.60)	<0.0001
**Neuropathy**	125 (18.0)	144 (15.4)	1.13 (0.86–1.48)	0.3909
**Fever with neutropenia**	29 (4.2)	102 (10.9)	0.37 (0.24–0.57)	<0.0001
**Diarrhea**	58 (8.3)	106 (11.3)	0.69 (0.49–0.98)	0.0374
**Pre-AE**	304 (43.7)	354 (37.9)		

2L: second-line; AE: adverse event; CI: confidence interval; CT: chemotherapy; IO: immunotherapy; OR: odds ratio

^1^Adjusted for first-line therapy (ref = CT), pre-existing AE (yes vs no), age, race, region, histology, smoking status, marital status at diagnosis, prior treatment of radiation, prior surgical treatment, and CCI (0, 1–2, and >2)

^2^Chi-square test

^3^Fisher’s exact test

## Discussion

In this real-world retrospective study in a VA population, approximately one-third of patients received 2L therapy and over 50% of these patients received CT as their 2L treatment. However, 2L IO therapy was associated with significantly better survival outcomes despite the IO group maintaining a greater number of comorbidities. Given the differences between clinical trial populations and patients seen in clinical practice, these findings represent valuable information that may be used to better understand the real-world effectiveness and safety, as well as patterns of use, of IO therapy.

In a similar real-world study of patients diagnosed with metastatic NSCLC and treated between 2012 and 2016, 40% of 1L patients received 2L therapy, a proportion similar to the 35% in our cohort [14]. Nadler et al. reported that among the 4,235 patients who had received 2L therapy, only 17% (n = 727) received IO, whereas 66% received CT and 17% received targeted therapy [14]. In our study, 43% of patients had received IO, and patients became more likely to receive IO than CT in more recent years. This increased IO utilization has similarly been observed previously [12, 15], suggesting that clinical practice is moving towards increased use of IO. The small portion of IO use pre-2015 may reflect off-label or clinical trial use, and the slight decrease in IO use we observed in 2017 is likely related to the introduction of IO as a 1L therapy at that time. The year of diagnosis and of treatment administration were consistent with US Food and Drug Administration approvals in both 1L and 2L in the VA setting.

Understanding the treatment decisions being made in the real world is important. Various factors inform 2L treatment decisions, including type and outcomes of 1L therapy, histology, physical functioning, age, comorbidity status, patient preference, and biomarkers. In our study, IO utilization was more prevalent in the West and Midwest of the US than other regions, indicating that there are geographic differences in prescription patterns. Patients who had received IO as 1L therapy were significantly less likely to receive IO as 2L therapy. Increasing age was associated with greater use of 2L IO therapy, and those with a high CCI score were significantly more likely to receive IO. This suggests that healthcare providers may recommend IO over CT in patients who are perceived to be more frail, likely in part because of the AEs associated with CT. Factors not typically captured in electronic health records, such as health-related quality of life and symptom burden, also play a major role in the type of, if any, 2L therapy received. In a Surveillance, Epidemiology and End Results-Medicare cohort of patients with stage IV NSCLC, there were no statistically significant associations between demographic characteristics and receipt of 2L IO [15]. Contrary to our findings, these authors found no association with treatment with IO and comorbidity score, and observed that squamous cell histology correlated with greater odds of receiving IO. We noted shorter treatment duration in the CT group compared to IO which could be due many factors including histology, regimen, symptom severity, and patient preferences, and optimal IO duration remains unknown.

The survival improvements associated with IO versus CT in our study are consistent with results from previous studies. In our study, patients who received IO had a median OS of 8.7 months compared with 7.0 months for CT. Other population-based studies have reported a median survival from 2L IO therapy initiation of 9.7 months, compared with 9.2 months for CT [14]. A recent study among Veterans receiving immune checkpoint inhibitors in the 2L setting observed a median survival of 9.9 months, and the HR for IO (i.e. pembrolizumab or nivolumab) versus CT (i.e. docetaxel) was 0.639 (95% CI 0.592–0.69) [8]. Also, our study finding is generally similar to a previous real-world study performed in the Flatiron Health database, which found that 2L IO improved median OS in patients with NSCLC by approximately 3 months, compared with 2L CT (17.5 vs 14.2 months from 1L initiation, respectively) [16]. Survival outcomes in our study fall within the range of that observed in clinical trials [17]. Additional research using the same time point for the beginning of OS assessment is required to confirm real-world OS for 2L NSCLC therapies [18]. Also, as IO utilization continues to increase it will be important to evaluate if and to what extent there is long-term survival benefit of IO utilization compared to CT.

HCRU was numerically lower for CT patients than for IO patients. However, on multivariable analysis, no significant difference was found between the treatment cohorts for any HCRU variable examined. Findings on this outcome in previous studies have been mixed. Clinical trial data have shown lower HCRU in IO patients than in CT patients in previously treated advanced NSCLC [19]. In other real-world studies, HCRU in NSCLC varied, depending on the country of treatment [20]. As expected, common AEs in the IO group were dyspnea, colitis/enterocolitis and anemia, and common AEs in the CT group were anemia, colitis/enterocolitis and nausea/vomiting. Studies have reported that gastrointestinal immune-related AEs such as colitis are known to occur in 30–50% of patients receiving immune checkpoint inhibitors and dyspnea in general is common in up to 50% of advanced cancer patients [21–23]. Previous research has found that hypothyroidism or pneumonitis is most common in patients treated with IO therapy [18, 24]. However, overall, the AEs observed align with those found to be common in other real-world studies of 2L NSCLC therapy [25]. As most studies focus on IO survival outcomes, the literature is scarce regarding HCRU and the prevalence of AEs in population-based studies of IO therapy.

A key strength of our study is the utilization of the most recent data available in the robust VA Oncology database, which is richly annotated with cancer diagnosis data as well as longitudinal data on the clinical care received within the VA healthcare system. These data are extremely useful for comparative effectiveness research and evaluating real-world treatment utilization patterns and associated outcomes. Also, relative to other studies, this cohort has a larger population of African American patients, who are often underrepresented in clinical trials. This study adds to the growing body of evidence of improved survival outcomes with IO use, as well as contributes findings on HCRU and AE occurrence in the 2L setting. However, our study also has some limitations. Rates of common AEs reflected ICD-9 and ICD-10 codes, in contrast to strict protocol-defined AEs, which may have contributed to the inconsistencies observed in clinical trials. Use of ICD-9 and/or ICD-10 codes to capture AEs implies that some of the AEs observed may not have been due to lung cancer or anticancer therapy. To minimize this possibility, we assessed AEs within a finite time period, post-treatment. However, this may have resulted in under-reporting of late-onset AEs. In addition, this study is unable to assess the severity of each AE. Pre-existing AEs were not excluded from the study but instead treated as independent variables. It is also important to note that duration of drug exposure was not adjusted for, which could have been the reason for the surprising findings of fewer AEs in CT patients than in IO patients. Treatment duration and pre-existing AEs likely contribute to post-treatment HCRU and the occurrence and severity of AEs.

It was not possible to capture patient performance status or biomarker status as the database lacked comprehensive and structured biomarker information. Therefore, it was not possible to assess treatment patterns by biomarker status. In particular, PD-L1 (programmed death-ligand 1) expression is associated with better outcomes than PD-L1 negative disease when treated with immune checkpoint inhibitors in metastatic NSCLC. Because of the lack of uniform gene mutation biomarker data, we used administration of targeted therapy as a proxy for EGFR, ALK, and ROS1 mutation status in order to exclude these patients from the study.

While we were able to assess which IO agents were administered, we cannot confirm whether they were indicated or used off-label. It was not possible to capture care received outside of the VA healthcare system, as some patients may have had other insurance through which they received medical care. As with all real-world studies, selection bias may have occurred because selection is primarily determined by provider recommendation and patient acceptance. For example, given that no randomization is involved in observational studies, clinicians may offer less aggressive therapy to sicker patients and/or sicker patients may decline more aggressive therapy because of their other illnesses. We accounted for potential comorbidities in the analyses, but there are likely other potential confounders that we were not able to measure.

## Conclusions

In this large retrospective real-world study of a Veteran population who primarily received first-line chemotherapy for advanced NSCLC, only 35% received second-line systemic therapy. Of these, 43% received second-line immunotherapy, which was associated with significantly longer overall survival than second-line chemotherapy despite these patients sharing more comorbidities than their chemotherapy counterparts. Patients who were older, had a higher Charlson Comorbidity Index score, lived in the West or Midwest regions, and did not receive first-line immunotherapy were more likely to receive second-line immunotherapy. Healthcare resource utilization was similar among the treatment groups, but more patients receiving immunotherapy had at least one adverse event, possibly due to extended duration of drug exposure among immunotherapy patients. Among patients treated with first-line chemotherapy and without immunotherapy contraindications, second-line immunotherapy should be considered. The increasing availability and indications for immunotherapy will likely increase the administration of second-line therapy to NSCLC patients.

## Supporting information

S1 Table Medications captured in each treatment group. (DOCX)

## List of abbreviations

1L: First-line
2L: Second-line
AE: Adverse event
CDW: Corporate Data Warehouse
CI: Confidence interval
CT: Chemotherapy
EGFR: Epidermal growth factor receptor
HCRU: Healthcare resource utilization
HR: Hazard ratio
ICD: International Classification of Diseases
IO: Immunotherapy
NSCLC: Non-small cell lung cancer
OR: Odds ratio
OS: Overall survival
PD-L1: Programmed death-ligand 1
SAS: Statistical Analysis Software®
SD: Standard deviation
SE: Standard error
SITC: Society for Immunotherapy of Cancer
US: United States
VA: Veterans Affairs
