# Supplementary material for: Health outcomes and healthcare resource utilization among Veterans with stage IV non-small cell lung cancer treated with second-line chemotherapy versus immunotherapy
Source: PLoS One. 2023 Feb 21;18(2):e0282020. doi: 10.1371/journal.pone.0282020 (PMC9942992; doi:10.1371/journal.pone.0282020)
Supplement: S1 Table — (DOCX) [file pone.0282020.s001.docx]

| **IO** | **CT** |
| --- | --- |
| Atezolizumab | Carboplatin |
| Durvalumab | Cetuximab |
| Nivolumab | Docetaxel |
| Pembrolizumab | Etoposide |
|  | Gemcitabine |
|  | Paclitaxel |
|  | Ramucirumab |
|  | Vinorelbine |
|  | Pemetrexed |
|  | Bevacizumab |

Supplemental Table S1. Medications captured in each treatment group
